# Low-Temperature Plasmas Improving Chemical and Cellular Properties of Poly (Ether Ether Ketone) Biomaterial for Biomineralization

**DOI:** 10.3390/ma17010171

**Published:** 2023-12-28

**Authors:** John P. Bradford, Gerardo Hernandez-Moreno, Renjith R. Pillai, Alexandria L. Hernandez-Nichols, Vinoy Thomas

**Affiliations:** 1 Polymer and Healthcare Material/Devices, Department of Mechanical and Materials Engineering, The University of Alabama, Birmingham, AL 35294, USA; jpb@uab.edu (J.P.B.); hgerardo@uab.edu (G.H.-M.); rrpillai@uab.edu (R.R.P.); 2 Department of Cellular and Molecular Pathology, Heersink School of Medicine, The University of Alabama, Birmingham, AL 35294, USA; aln835@uab.edu; 3 Center for Free Radical Biology, The University of Alabama, Birmingham, AL 35294, USA; 4 Department of Physics, Center for Nanoscale Materials and Bio-Integration (CNMB), The University of Alabama, Birmingham, AL 35294, USA

**Keywords:** PEEK, bioactive surface, plasma modification, bone implant, biomineralization

## Abstract

Osteoblastic and chemical responses to Poly (ether ether ketone) (PEEK) material have been improved using a variety of low-temperature plasmas (LTPs). Surface chemical properties are modified, and can be used, using low-temperature plasma (LTP) treatments which change surface functional groups. These functional groups increase biomineralization, in simulated body fluid conditions, and cellular viability. PEEK scaffolds were treated, with a variety of LTPs, incubated in simulated body fluids, and then analyzed using multiple techniques. First, scanning electron microscopy (SEM) showed morphological changes in the biomineralization for all samples. Calcein staining, Fourier transform infrared spectroscopy (FTIR), and X-ray photoelectron spectroscopy (XPS) confirmed that all low-temperature plasma-treated groups showed higher levels of biomineralization than the control group. MTT cell viability assays showed LTP-treated groups had increased cell viability in comparison to non-LTP-treated controls. PEEK treated with triethyl phosphate plasma (TEP) showed higher levels of cellular viability at 82.91% ± 5.00 (*n* = 6) and mineralization. These were significantly different to both the methyl methacrylate (MMA) 77.38% ± 1.27, ethylene diamine (EDA) 64.75% ± 6.43 plasma-treated PEEK groups, and the control, non-plasma-treated group 58.80 ± 2.84. FTIR showed higher levels of carbonate and phosphate formation on the TEP-treated PEEK than the other samples; however, calcein staining fluorescence of MMA and TEP-treated PEEK had the highest levels of biomineralization measured by pixel intensity quantification of 101.17 ± 4.63 and 96.35 ± 3.58, respectively, while EDA and control PEEK samples were 89.53 ± 1.74 and 90.49 ± 2.33, respectively. Comparing different LTPs, we showed that modified surface chemistry has quantitatively measurable effects that are favorable to the cellular, biomineralization, and chemical properties of PEEK.

## 1. Introduction

Poly (ether ether ketone) (PEEK) has multiple properties, including mechanical properties, that make it a desirable material for several orthopedic applications; however, PEEK on its own lacks the bioactivity [1,2] necessary for successful osseointegration. This lack of bioactivity limits its application for use in bone implant technologies. This is due to its inability to form calcium phosphate (CaP) when implanted in vivo, which results in poor anchoring of the bone implant. For successful osseointegration, the implant needs surface bioactivity to promote bone repair as well as adequate mechanical properties [3,4]. Challenges in efficacious bone implant technologies are multi-faceted. With multiple areas identified for improvements, surface engineering of bone grafts/implants has become an increasingly appealing proposition.

Incorporating bone minerals (calcium phosphate or bio-glass) was a sought strategy. One group, has previously reported the enhancement of mechanical properties of the synthetic polymer polycaprolactone (PCL) upon the addition of hydroxyapatite [4]. Ye et al. showed the direct relationship between the quantity of bio-glass content in 3D printed hydrogel and mechanical compressive strength. The cell viability, at all time points, peaked at 10% bio-glass and decreased with an increasing amount of bio-glass in the hydrogel [5]. This demonstrates that, while the quantity of bio-glass material and compressive strength had a directly proportional relationship, the cell viability had an inverse relationship to the scaffold functionality. This demonstrates the challenge in striking a balance between mechanical properties and the biocompatibility of any scaffold. Fragal et al. used wet chemistry to modify cellulose nano-whiskers, which caused an increase in hydroxyapatite mineralization on the surface of the material [6]. In that study, a multi-step synthesis was performed, resulting in an amine modified scaffold that was then immersed in a weak calcium chloride solution before immersion in simulated body fluid (SBF). Hydroxyapatite was effectively biomineralized on functionalized nano-whiskers and demonstrated a cellular viability synonymous with hydroxyapatite; however, this process took several days and required intensive purification for both functionalization and surface` treatment of the nano-whiskers.

**Figure 1:**
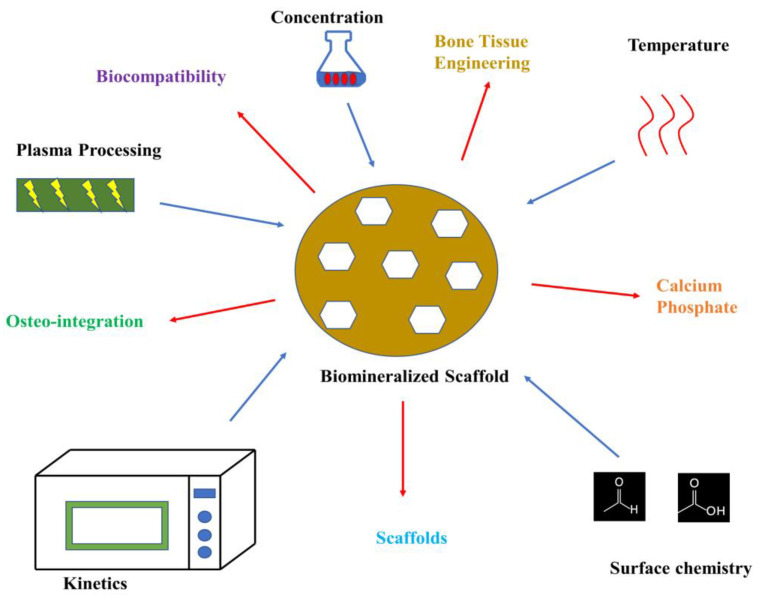
Schematic illustrating varied factors and applications revolving around biomineralized PEEK. The blue arrow pointing to the scaffold indicates some of the experimental variables changed to modify the substrate, while the red arrows show results and applications received from the process.

Several bioactive molecules are key in the bone remodeling process. Inflammation of implant site, immunogenicity, and instability of biomolecules themselves are just a few of the current challenges in BTE [7,8,9]. Several of the biomolecules, and the corresponding polymer they are often used with, have been extensively studied, for these applications. PDLA [10,11], BMP-2 [12,13] PDGP [14] TGF-B [15], and PLGA [16,17] are just a few of the polymers and biomolecules being used to addressed various problems in BTE. Low-temperature plasma offers a solution to modifying the surface chemistry of a polymeric scaffold, acting as a vehicle to transport these bioactive molecules to the surface of the polymer. This technique’s flexibility is demonstrated by the variety of applications, including surface modification [18], etching [19], modification of surface morphology [20], changing surface energy of a material [21], and others. Overall, several techniques, methodologies, and concepts can be implemented to optimize the biomineralization process. Figure 1 shows a schematic of some of the distinct factors that can be used to increase biomineralization on a scaffold and varied applications that occur as a result.

The ability to increase the cell viability and bioactivity of polymeric biomaterials using LTP, where bulk properties of the scaffold are unaffected, is well documented [22]. This can effectively resolve the issues of an inverse relationship between mechanical properties and cell viability that Ye et al. observed. Plasma processing is a quick single-step surface processing technique that can enhance the biomineralization of CaP in simulated body fluid in vitro, allowing for the multi-phased morphology of CaP in body-like conditions. This is optimal because hydroxyapatite makes up only one of the many compositional morphologies reinforcing sections of bone. SBF has been experimentally used to quantitatively analyze the morphological characteristics expected to be on the surface of treated scaffolds prior to implantation [23].

PEEK applications in orthopedics continue to grow, with just a couple being in fracture fixation [24,25,26] and commercialized dental implants such as VESTAKEEP PEEK. PEEK is one of several polymers that has been investigated for scaffold application in BTE, some of which include PCL [27,28], Polyurethane [29], and PLA [30,31,32]. PEEK surface modification to enhance bioactivity has been documented using plasmas, including poly acrylic acid [1] and a water argon gas mixture [2]. Studies reported that some forms of surface modification of PEEK improved its bioactivity, but further optimization of this polymeric surface is necessary for functional use in bone implants [1,2,22]. LTP utilizing phosphate functional groups is one application used in creating thin films and coating preparations [33,34,35,36]. Despite the advantages of surface functionalization with the chemical moiety PO_4_^3−^, we are not aware of studies that have leveraged the readily available LTP technology to stimulate the biomineralization of CaP on materials. Our study hypothesized that using LTP technology to modify PEEK with negatively charged phosphate groups, obtained through triethyl phosphate (TEP), would provide a higher quantity of complexing CaP upon immersion of the modified scaffold in SBF. CaP bonding is electrostatic [37]. Mechanistically, the PO_4_^3−^ ion, provided by the TEP plasma treatment, will cause immobilization of the Ca^2+^ ion. Electrostatic bonding of PO_4_^3−^ will occur, with the ionic Ca^2+^ subsequently causing nucleation forming CaP on the surface, which will result in increased bioactivity of PEEK [38].

This study aims to perform the following: (1) explore a novel but robust method to supply phosphate functional groups to the surface of PEEK; (2) compare the phosphate moiety with other chemical functionalizations known for increasing CaP biomineralization on PEEK in SBF conditions; (3) examine cell viability and (proliferation) of osteoblast-precursor cells on PEEK with the differing plasma modifications; (4) validate findings by varying kinetic conditions to demonstrate chemical trends with this surface processing technique. In this study, TEP is used to supply phosphate functional groups to PEEK via LTP and this is then compared with nitrogen functional groups supplied by an ethylene diamine (EDA) plasma, which has been recently used to enhance biomineralization on a PLA (polylactic acid) 3D printed scaffold [39], and also with the use of a methyl methacrylate (MMA) plasma, which in prior studies has resulted in increased hydrophilicity of a polymeric scaffold through oxidation [40].

## 2. Materials and Methods

### 2.1. Materials

Ethylenediamine (EDA), triethyl phosphate (TEP), and methyl methacrylate (MMA) were purchased from Sigma-Aldrich (St. Louis, MO, USA). PEEK 450G was purchased from Victrex. Potassium phosphate dibasic trihydrate, sodium chloride, sodium sulfate, and sodium bicarbonate were all purchased from Arcos Organics. Tris (hydroxymethyl) aminomethane and calcium chloride were obtained from Alfa Aesar (Haverhill, MA, USA). A 1 step PNPP substrate solution was purchased from Thermofisher Scientific (catalog number 37621). The Invitrogen CyQUANT cell proliferation assay kit was purchased from Thermofisher Scientific (catalog number V13154) (Waltham, MA, USA). All materials were stored according to the manufacturer’s instructions.

### 2.2. Scaffold Formation and Biomineralization Process

A total of 2 g of PEEK pellets were placed in between 2 aluminum cylinders and pressed into a circular scaffold at 354 °C and held for 1 min for the scaffolds to obtain a diameter of 8 mm. The cylinders were then removed and allowed to cool for 3 min before the scaffold was removed from between the cylinders. The scaffold was cut into several pieces, dimensions of 3 × 3 mm (about 0.12 in) and placed into the Harrick PDC-001 (Harrick Plasma Inc., Ithaca, NY, USA) and then treated with different plasmas on both sides of the scaffold for 30 min at 0.400 Torr. The PEEK materials were immediately placed in SBF solution, prepared according to ISO (International Standardization Organization) standard 23317 [41], scaled to a 1.5× concentration, and incubated for 18 days, avoiding the problems of hydrolysis, and maintaining the integrity of the apatite layer [42], where the solution was changed every 72 h.

#### Accelerated Biomineralization of Scaffold

Discs of 5 and 8 mm diameter were made from the plasma-treated PEEK, using carbide punches of the appropriate diameter, and placed in 10 mL of 1.5× SBF. They were treated under 1240 W microwave irradiation for 4 min, where the composition of the 1.5× SBF is described in our prior work [43].

### 2.3. Characterization of Biomineralized PEEK

#### 2.3.1. Chemical and Biomineralization Characterization

##### Calcein Staining

A total of 100 mg of each mineralized PEEK sample was lightly washed in DI water. Each sample was immersed in a pH 10.3 ± 0.1 1 M NaOH/DI water solution and sonicated for 5 min to dissolve the CaP on the PEEK scaffold. A total of 98 µL of the supernatant was mixed with 2 µL of a 0.02 M calcein solution. The solute of each sample was placed on a well plate and measured with the Ultra-LUM ultra-Cam G6 PN: 910-4110-10 digital imaging system. The camera used to capture the fluorescent images had 12 megapixels with an F/1.7 50 mm lens. ImageJ determined the pixel intensity from the fluorescence of each sample to quantify the number of pixels taken out from raw RGB images. Microsoft Excel for Mac version 16.76 was used to determine the standard deviation from the pixel size of each sample and to perform a Student’s *t*-test.

##### Fourier Infrared Spectroscopy (FTIR)

A Bruker Alpha FTIR system was used to identify the chemical functional groups of the biomineralized surface via ATR mode. The spectrum measured ranged from 400 to 4000 cm^−1^ and 64 scans of each scaffold were performed for better peak resolution.

##### X-ray Photoelectron Spectroscopy

PHI 5000 Versaprobe imaging was used to perform XPS (Physical Electronic Inc., Boston, MA, USA). A monochromatic focused X-ray with an Al k-alpha source with a 25 W power and 100-micrometer spot size. Analysis was performed with the Multipak v9.0 software. The control sample was one where no plasma treatment was performed on the scaffold, but was submerged in SBF for 18 days. Due to the scaffold formation method, small quantities of metallic compositions were detected during XPS characterization, but these were excluded from the reported data.

#### 2.3.2. Morphological Analysis

##### Scanning Electron Microscopy (SEM)

SEM was used for analyzing the surface morphological changes of the PEEK scaffold upon mineralization of the CaP. A Quanta FEG 650 Scanning Electron Microscope Quanta FEG 650 from FEI, Hillsboro, OR, USA) was used in high vacuum mode. An Au-Pd coating was sputtered onto the polymer due to the non-conductive nature of PEEK. For all samples, 10 KV at a spot size of 4 was used.

##### Water Contact Angle (WCA)

Static water contact angle (WCA) was used to examine the hydrophilicity of the PEEK surface with differing plasma treatments. A syringe was used to place a 7 µL water droplet on the surface of each scaffold. Free2X Webcam recorder software captured the image and ImageJ software 1.53 was used to analyze the material. Six measurements were used for each group, with averages reported with standard deviation.

##### Surface Roughness Measurement

Keyence microscope model VHX-6000 was used to perform surface roughness measurement on the PEEK material before incubation into SBF. The profile length used on all samples was 500 µm. The Ra values were collected for each sample with the standard deviation reported (*n* = 7 for each sample).

#### 2.3.3. Biological Characterization

##### Alkaline Phosphatase (ALP) Absorbance

A 1 step P-nitrophenyl phosphatase disodium salt (PNPP) was used to measure the absorbance of ALP. The manufacturer’s procedure was used to perform this characterization.

##### The 3-(4,5-dimethylthiazol-2-yl)-2,5-diphenyltetrazolium Bromide (MTT) Cell Assay

An Invitrogen CyQUANT cell proliferation assay kit was used to detect cell proliferation of the MG-63 cell line osteoblastic-like cells with a fibroblast morphology. Using a 96-well-plate format, 30,000 cells/well were seeded and the manufacturer’s procedure was followed.

## 3. Results and Discussion

### 3.1. Wettability of Plasma-Treated PEEK

**Figure 2:**
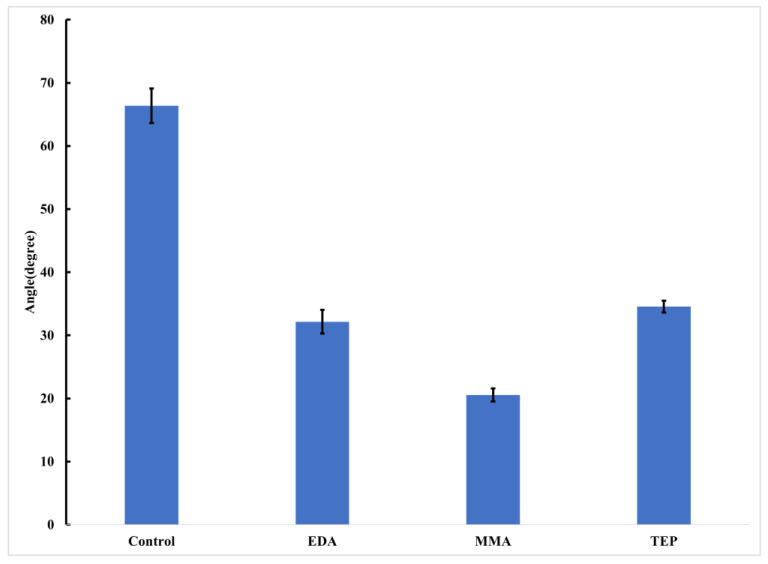
Water contact angle of PEEK samples before biomineralization, where the standard deviation of each is shown by the error bars and N = 6 for each sample.

The experiment was designed to compare both the biomineralization effect on the PEEK surface and the cellular viability demonstrated by varying LTP. Utilizing different LTPs causes different functional groups to appear on the scaffold, but this also affects the wettability of the PEEK surface. The wettability of PEEK may directly affect osteoblastic adhesion, and as it has been shown, that decreased wettability of a scaffold surface impedes osteoblastic attachment on polymeric surfaces [44]. Water contact angle measurements showed an increase in wettability in all treatment samples compared to the control samples (Figure 2). The significant increase in nitrogen functionalization explains the wettability increase in EDA. The TEP sample was originally expected to increase the contact angle of the neat sample due to the increase in phosphate chemical functionalization. However, there is a decreased contact angle due to phosphate bonding with additional oxygen groups forming the PO_4_^3−^ ion, and the increased surface roughness from the plasma treatment [45]. A probable cause of the increased wettability of the MMA plasma-treated peak can be attributed to the significantly higher oxygen composition in comparison to the control PEEK scaffold shown in the XPS data in Table 1 and Table 2.

**Figure 3:**
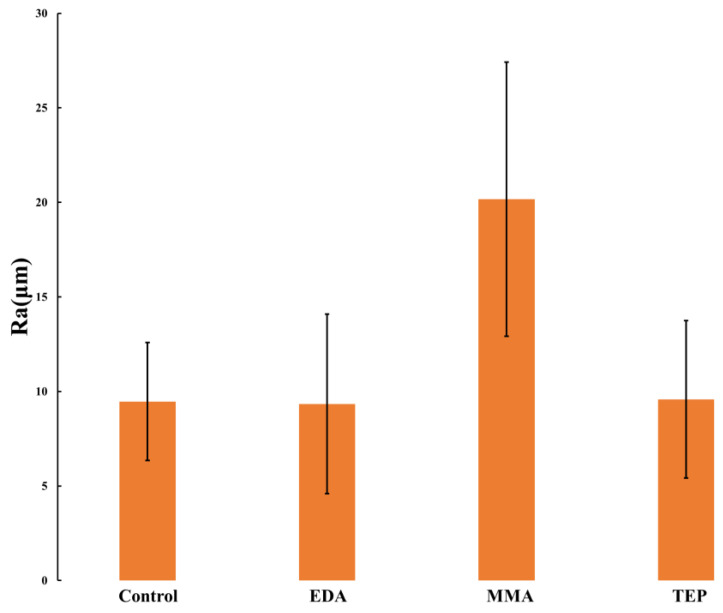
Ra value of surface roughness measurement of plasma-treated PEEK. The standard deviation is shown with error bars where *n* = 7 for each sample.

In addition to the increased oxygen composition of the MMA-treated PEEK sample, wettability is affected by the differing surface roughness for the PEEK sample resulting from the plasma treatment [40]. It was expected that MMA-treated PEEK would have a higher WCA than the rest of the PEEK samples, including the neat PEEK, due to its polymer counterpart being hydrophobic. Wettable materials are materials with a water contact angle < 90°. The surface wetness of a material can be increased via increasing the surface roughness. Analyzing the water contact angle together with surface roughness allows for a clear understanding of the effect of both the functional group change and surface roughness on wettability. In general, plasma treatment of polymeric samples increases the surface roughness of the material, and amongst wettable materials, this increased surface roughness causes increased wettability. Scaffolds treated with EDA did not show increased surface roughness, unlike all other plasma-treated scaffolds; however, nitrogen-attached functional groups, from plasma treatment with EDA, caused increased wettability for those scaffolds [46,47,48]. The chemical functionalization provided by LTP along with the surface roughness explains why all plasma-treated samples in this study had a significant decrease in water contact angle [49,50,51]. The Ra values of the different samples are displayed below in Figure 3.

### 3.2. Chemical Characterization of Biomineralization

**Figure 4:**
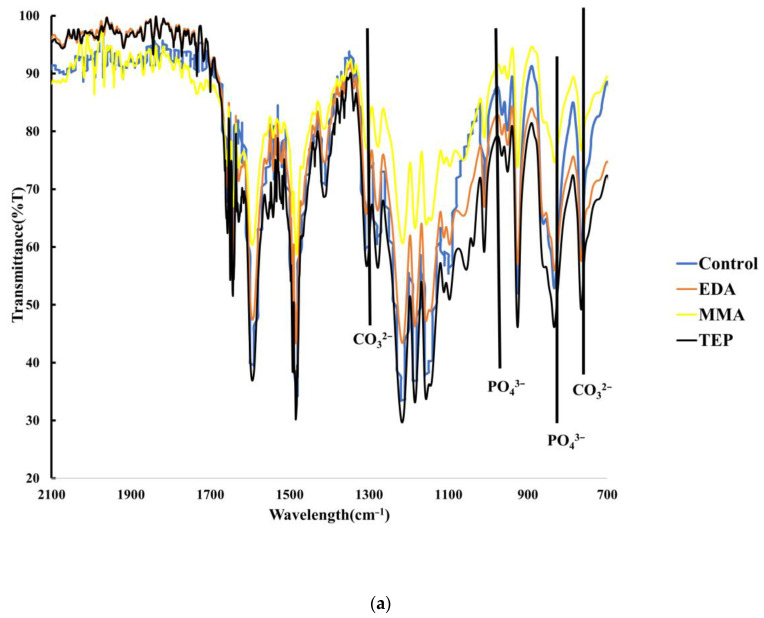

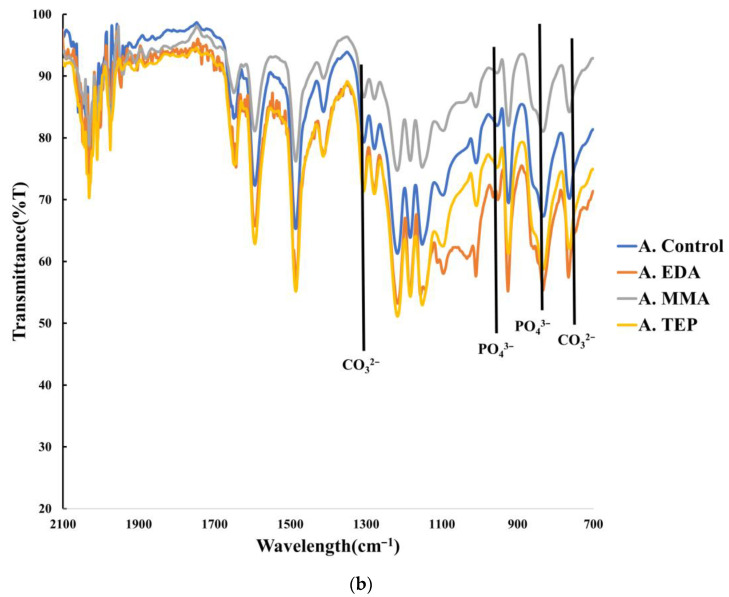
(**a**) FTIR of all biomineralized PEEK samples exemplifying the carbonate and phosphate peaks. (**b**) FTIR spectra of accelerated biomineralized samples illustrating carbonate and phosphate peaks.

Chemical characterization of the biomineralized PEEK samples was conducted using FTIR. Figure 4a and Figure 4b shows the significant increase in this PO_4_^3−^ ion in the FTIR by the increased intensity of the phosphate ion peaks identified at approximately 940 and 1040 cm^−1^ for the P-O and P=O stretching, respectively [52,53]. Using a common scale to overlay the FTIR data allowed us to measure the quantification of CO_3_^2−^ and PO_4_^3−^ by directly comparing the size of the peaks.

The accelerated biomineralized PEEK biomaterial showed similar chemical shifts as the material incubated for 18 days. The kinetic increase of the biomineralization using microwave irradiation resulted in a more amorphous CaP than the material incubated for 18 days, as would be expected. Figure 4b, compared to Figure 4a, showed broader spectral peaks, comparatively not as sharp, attributed to the less crystalline mineralization performed.

Querido’s experiment explains the relationship between crystallinity and FTIR peak appearance. He used the Pearson correlation coefficient and analyzed the correlation between the crystallinity of the CaP using the apatite peak found at about 1015 cm^−1^, showing that sharper and more intense peaks at this point signify higher levels of crystallinity of the CaP formation [54]. Thus, the FTIR spectra behaved in a manner predicted by the prior literature.

XPS showed a substantial increase in the oxygen composition of the TEP PEEK sample, as shown in Table 1. The XPS further showed that the phosphate functional groups were more effective in increasing the compositional percentage of calcium appearing on the surface versus either the EDA or MMA plasma-treated group exhibited in Table 2. Accelerated mineralization showed varying results concerning the compositional makeup of CaP mineralization (Table 3). Surprisingly, during accelerated mineralization, both TEP and MMA had significant PO_4_^3−^ take up in the sample and showed a significant decrease in Ca^2+^. These results also correlate to the FTIR data, which clearly show increased levels of PO_4_^3−^ for the A. MMA and A. TEP samples versus the MMA and TEP samples in Figure 4a,b.

**Figure 5:**
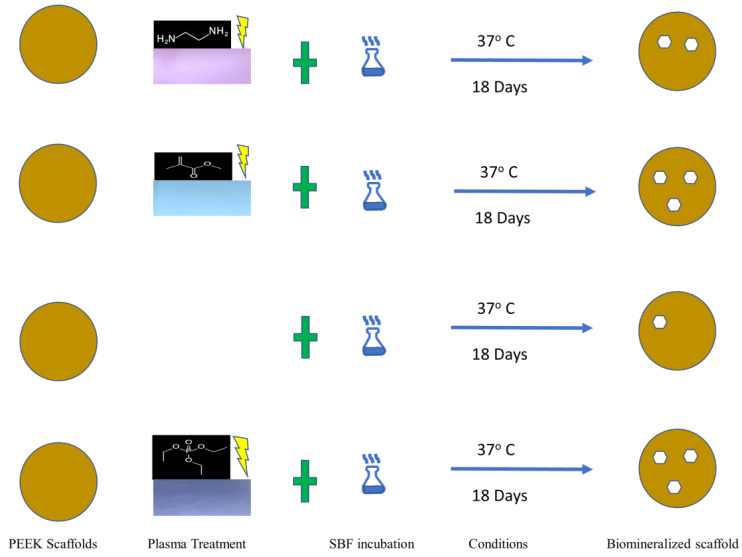
Schematic illustrating the biomimetic process utilized in this study, which shows the varying degrees of biomineralization based on the specific surface plasma treatment, or lack thereof, that occurred.

Our previous work operated on the notion that both surface chemical modification and topography etching are instrumental in optimizing the conditions for CaP to be mineralized in biomimetic conditions. LTP allows the top few nanometers of the polymeric scaffold to be etched on the top. This positively affects the cellular interactions with the surface of the polymer and increases the quantity of biomineralization [55]. This further substantiates the increase of surface area, resulting from LTP treatment, which can contribute to both the roughening of the polymeric surface morphology and the chemical moieties availability, where both can aid the formation of CaP [56], as demonstrated in Figure 5.

**Figure 6:**
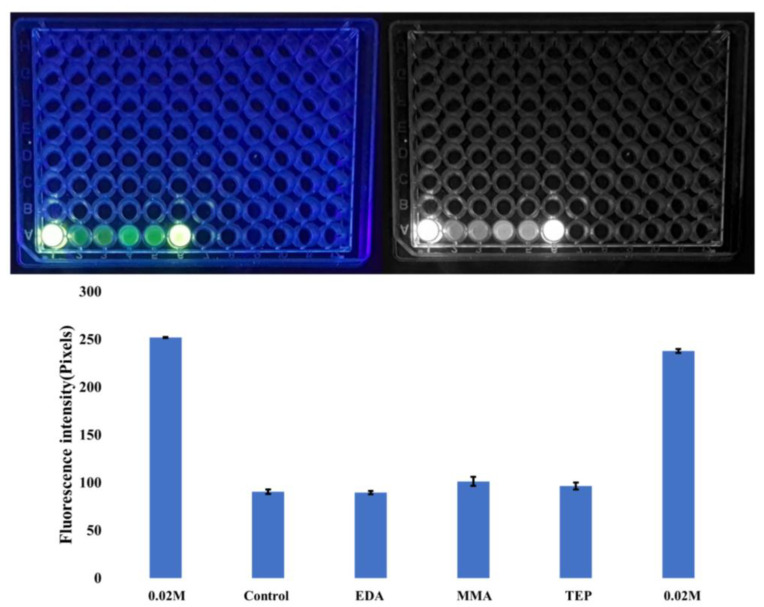
Fluorescence intensity of calcein staining with standard deviation shown by error bars where N = 6 for each sample. The fluorescent image of the 64 well plates show each of the 6 calcein stained samples, in direct coordination with and referred to in the graph.

Cotrut et al. compared techniques used to increase the surface roughness, and their results showed that increased surface roughness enhances apatite mineralization ability and increases osteoblastic in vitro response [57]. Negatively charged particles have been used in previous studies to enhance the biomineralization of CaP on scaffold surfaces, which explains why this study takes a dual approach using a singular technique in increasing the bioactivity of PEEK [58,59]. FTIR results in Figure 4a and Figure 4b and the calcein fluorescence intensity difference shown in Figure 6 demonstrate increased levels of mineralization that come as a byproduct of the roughened morphology and increased chemical functional groups.

The increase in intensity of the fluorescence indicates more CaP bound by calcein. Due to the SBF immersion process, loose salts may be present on the scaffold’s surface; therefore, the materials are washed to mitigate background noise in the signal. The byproduct resulting from CaP is bound to the surface of the PEEK and is responsible for the resultant detected levels of fluorescence. It is well documented that greater levels of CaP allow for superior surface/cellular interface interaction. Additionally, optimizing the surface for the body to naturally mineralize the phase of CaP enhances the bioactivity of PEEK. Thus yields a better cellular environment with fewer processing steps needed for the scaffold [60].

### 3.3. Biological and Morphological Characteristics of PEEK Samples

**Figure 7:**
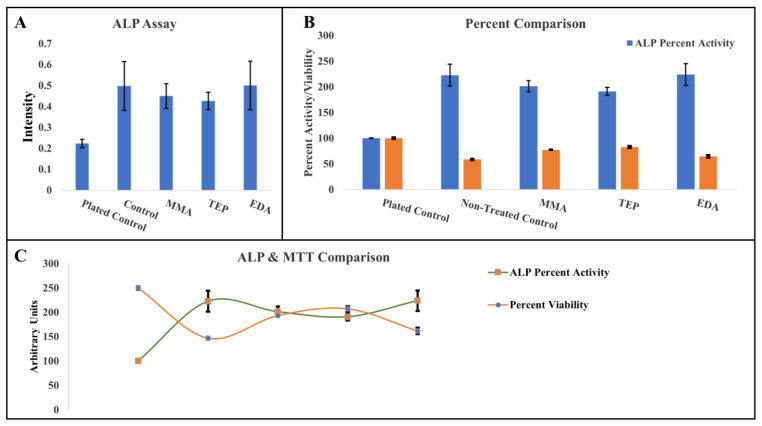
(**A**) Intensity display of ALP assay amongst all samples. (**B**) Display of MTT viability in terms of percentage. (**C**) Relationship in the MTT and ALP analyses.

The cell viability showed a statistically significant increase in all LTP-treated groups in comparison to the untreated control PEEK scaffold shown in Figure 7b. TEP showed a more than 30% increase in cellular viability percentage than the control at (82 ± 5)%, whereas MMA and EDA showed a statistically significant increase in viability at (77 ± 8)% and (68 ± 5)% compared to the control at (59 ± 6)%. Standard deviations varying by less than 10% for all characterizations demonstrated the consistency of the method throughout all groups. The ALP assay showed the control PEEK sample having higher expressions of the ALP enzyme when compared to all other plasma-treated samples. All samples showed significantly higher expression than the plated control indicated in Figure 7a. Rapid differentiation of osteoblastic cells was not observed in the LTP-treated PEEK samples. However, increased osteoblast viability with decreased osteogenic differentiation is a common outcome for apatite layered materials [43]. With the other chemical characterization showing higher levels of CaP surface mineralization in this study, mineralization may not occur for several days in the SBF in vitro [58,61,62,63,64]. Several studies have shown that the process of mineralization may take time in SBF for mineral formation. It was believed that cell viability would increase with plasma treatment due to the increased surface area. ALP is considered an early gene indicator for apatite formation and the weak expression despite strong apatite formation can be explained. Zhou’s group performed gene expression on PLA bionanocomposites, and similar to this study, the expression of ALP, in their PLA bionanocomposites, was reduced despite an increase in apatite. The group listed several possibilities for this to happen but could not conclusively state why [65].

**Figure 8:**
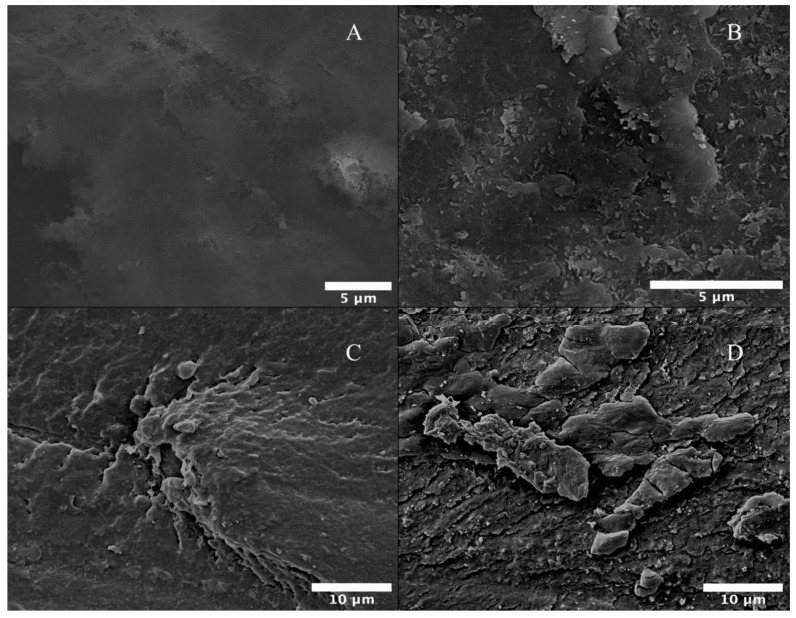
Representative SEM images showing the morphology of biomineralized samples. (**A**) Control samples at 5000× magnification. (**B**) EDA sample performed at 10,000× magnification. (**C**) MMA sample at 3000× magnification. (**D**) Phosphate sample at 3000× magnification.

Multiple formulations of SBF biomineralization have been used in past studies, however, this study used the method proposed by Kokubo as it is the most utilized strategy in apatite biomineralization in SBF conditions. He stated that there is a direct time correlation between the apatite formation on the surface of a material and the time it takes for a material to bond to living bone. He concludes that the degree of apatite formation on a material predicts the in vivo bioactivity of that material [66,67,68,69]. Apatite mineralization, which can be tested by SBF immersion, is a logical precursor to both in vitro and in vivo studies, both of which consume time and resources [70]. Additionally, mineralization formation within the physiological extracellular matrix (ECM) allows yields with comparably better cellular responses than using a scaffold with the CaP surface coating present before implantation of the scaffold [71]. This will help decrease the inflammation response of the body due to the fibrous capsulation of the scaffold upon implantation, and this same inflammation response dictates the biocompatibility of the biomaterial being used [72]. The quantity, morphology, mechanical properties, and composition of mineral formation on scaffolds used in body-like conditions are critical in optimizing biomineralization for increased bioactivity of PEEK. The mechanical properties of the scaffold will affect cellular adhesion onto the material, hence material selection and maintaining mechanical properties throughout physiological healing are of primary concern in tissue engineering. Additionally, osteoblastic adhesion can be modulated using the mineralized CaP on the polymer surface [73]. The SEM images in Figure 8 show the significant increase of CaP on the surface of the treated PEEK compared to that of the control scaffold. Nucleation points created through the LTP treatment allowed the morphology of the particulates to form, as shown in the higher magnification images (Figure 8b).

**Figure 9:**
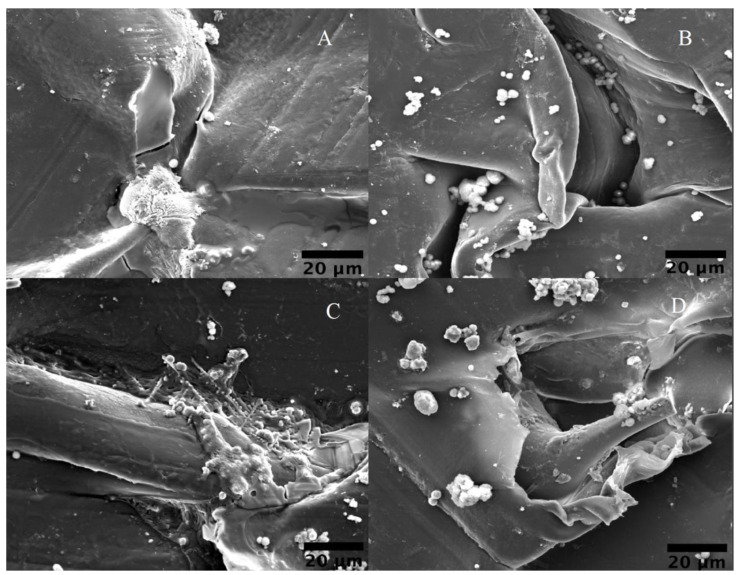
Representative SEM images displaying morphological features of the biomineralized samples; (**A**) 3000× magnification of A. control, (**B**) A. EDA images at 3000×, (**C**) A. MMA at 3000× magnification, (**D**) A. TEP images at 3000× magnification.

The coarse morphology seen in Figure 8b–d results from the significant amount of mineralization that occurred from plasma treatment. The CaP particle agglomeration, in the biomimetic biomineralization shown in Figure 8, differs vastly from the accelerated biomineralized samples, as seen in Figure 9. SEM showed the kinetics of how mineralization affects both the morphology and particle agglomeration on the surface of the polymer, but chemical indicators, extracted from comparative FTIR and XPS data, showed the same thermodynamic formation [74].

## 4. Conclusions

Novel LTP treatments were discussed and the resulting biomineralization observed on the PEEK surface evaluated. After scaffold formation, this one-step surface treatment technique allowed for effective surface roughening and surface functionalization of those scaffolds. Furthermore, this technique allows for the modification of a material’s surface, without affecting the bulk material properties. This allows for controlled optimization of a material’s biocompatibility, which can be found using scaffold-implanted bone tissue engineering applications. Several different LTPs are often used to modify a material’s surface in a manner that would be conducive for biomineralization. FTIR, XPS, and calcein staining showed increased levels of both carbonate and phosphate resulting from TEP plasma treatment. Generation of phosphate groups across the surface of PEEK using TEP resulted in an increase of CaP biomineralization on the surface of the PEEK material. This CaP biomineralization led to an increase in cellular viability and proliferation compared to all other LTP treatment methods. Although the TEP plasma-treated group demonstrated the most significant increase in biocompatibility, all plasma-treated groups showed significantly higher levels of cellular viability and proliferation in comparison to the untreated control scaffold. This study has major implications for the future of PEEK in BTE and the advantage of the one-step surface treatment method of LTP; additionally, a possible methodology to decrease implant-associated inflammations was proposed. Future research would test the PEEK implant’s bio-integration in an animal model for exploring the biomineralization efficacy in vivo.

## Figures and Tables

**Table 1:** XPS elemental composition of plasma-treated samples.

	C1s	O1s	P2p	N1s
Neat	77.5	18.9	0	2.6
TEP	47.9	41.3	10.2	0.6
MMA	63.0	30.9	0	0
EDA	73.6	10.2	0	14.0

**Table 2:** XPS elemental composition of biomineralized samples.

	C1s	O1s	P2p	N1s	Ca2p
Control	87.1	8.4	0	0	0.9
TEP	57.0	28.5	1.8	4.2	1.5
MMA	86.8	10.7	0	1.2	0.8
EDA	75.0	15.8	0	1.9	1.2

**Table 3:** XPS elemental composition of accelerated biomineralized samples.

	C1s	O1s	P2p	N1s	Ca2p
A. Control	73.2	20.6	0.7	2.1	1.0
A. TEP	76.7	20.6	2.1	0	0.6
A. MMA	60.8	29.7	3.4	4.8	1.3
A. EDA	78.8	16.2	0	2.6	0

## Data Availability

All necessary data has been presented in this article. Any further information can be made available upon any request made to the authors.
